# Socioeconomic Inequality and Spatial Distribution of Drug Abuse Mortality in Iran: A National Study in 2020

**DOI:** 10.34172/jrhs.11568

**Published:** 2026-02-21

**Authors:** Hoda Arabzadeh, Faezeh Heidari, Seyed Saeed Hashemi Nazari, Fatemeh Shahbazi

**Affiliations:** ^1^Department of Epidemiology, School of Public Health, Hamadan University of Medical Sciences, Hamadan, Iran; ^2^Students Research Committee, School of Public Health, Hamadan University of Medical Sciences, Hamadan, Iran; ^3^Department of Epidemiology, School of Public Health and Safety, Shahid Beheshti University of Medical Sciences, Tehran, Iran; ^4^Prevention of Cardiovascular Disease Research Center, Shahid Beheshti University of Medical Sciences, Tehran, Iran; ^5^Modeling of Noncommunicable Diseases Research Center, Hamadan University of Medical Sciences, Hamadan, Iran

**Keywords:** Substance use, Addiction, Inequality, Mortality, Iran

## Abstract

**Background:**

Drug abuse is a public health problem that leaves morbidity, disability and premature mortality in the society. This study investigated the epidemiological features, socioeconomic disparities, and geographic distribution of drug abuse-related deaths across Iran in 2020.

**Study Design:**

An ecological study.

**Methods:**

Data on drug abuse deaths were obtained from the Iranian Legal Medicine Organization (LMO). The Theil index, between-group variance (BGV), concentration index, and concentration curve (CC) were used to assess mortality inequality. Finally, spatial inequality was analyzed using Anselin’s Local Moran’s.

**Results:**

In 2020, the drug abuse mortality rate was 49.91 per million people in Iran. Opium (24.89%), methamphetamine (21.23%), and heroin (16.58%) were the most common substances involved in this respect. High-high clusters, including Hamadan, Markazi, Lorestan, and Ilam, had high drug abuse death rates and were surrounded by similar provinces. The concentration index of 0.10 indicated higher drug-related deaths in provinces with a higher human development index (HDI). Moreover, the Theil index and BGV revealed considerable regional inequality in drug abuse mortality rates.

**Conclusion:**

Overall, drug-related deaths in Iran predominantly affected males, particularly in the 30–39 age group. Victims had low educational attainment and were self-employed. Provinces with lower HDIs, such as Kermanshah, Hamedan, and Lorestan, had the highest mortality rates. Thus, these regions need targeted prevention and treatment. Addressing the links between substance use and suicidal behavior requires integrated mental health and addiction treatment services. In addition, policymakers should prioritize educational, preventive, and treatment programs in high-risk areas.

## Background

 Substance use disorder (SUD) is a complex psychiatric and social condition that involves the harmful use of psychoactive substances, including illicit drugs (e.g., heroin, opium, and cannabis).^1^ It leads to serious psychological and physiological dependence, resulting in adverse physical, mental, and social consequences that may lead to disability or premature death.^2,3^

 Despite global efforts to reduce substance abuse, illicit drug use remains a growing public health challenge worldwide. The burden is pronounced among young populations, especially in developing countries, where the social and economic impacts of drug addiction can undermine health and welfare systems.^4^ According to the Global Burden of Disease Study (GBD), drug use disorders (DUDs) cause about 5% of all deaths and 8.5% of total disability-adjusted life years (DALYs) lost among individuals aged 15–49 years worldwide. Drug use–related DALYs noticeably rose from 2000 to 2016, with the largest increase (36%) in cocaine and amphetamine use disorders.^5^ Based on the 2021 GBD Study, DUDs are among the top 20 causes of DALYs for the 10–49 age group globally. Over 296 million people used drugs in 2021, with 39.5 million diagnosed with DUDs, representing a 45% increase over ten years. Nonetheless, only one in five receives treatment, with growing geographical disparities. A 2020 United Nations Office on Drugs and Crime report estimated 284 million drug users aged 15–64. DUDs severely harm physical and mental health and contribute to public health crises, including infectious diseases (e.g., acquired immune deficiency syndrome).

 Iran faces major substance abuse challenges due to its proximity to drug-producing neighbors, Afghanistan and Pakistan.^6^ According to official reports, over two million people in Iran are addicted to substances, with the prevalence having tripled over the past two decades.^7^ Substance-related mortality ranks as the second leading cause of suspicious deaths after traffic accidents in the country.^8^ The economic burden of drug addiction in Iran exceeds five million dollars annually.^9^

 Epidemiological studies in Iran demonstrate that drug-related mortality is the highest among young, unmarried males with low education and income.^10-12^ Despite the growing body of evidence, spatial disparities in drug-related mortality and their geographic distribution have not been sufficiently studied so far. Understanding these disparities is crucial to developing effective public health interventions that target high-risk areas and mitigate the negative consequences of substance abuse.

 Socioeconomic inequality and spatial disparity are deeply intertwined in the context of Iran. Regions with a lower socioeconomic status face greater risks (e.g., unemployment, poverty, and poor access to services), leading to higher substance abuse and mortality. Therefore, analyzing spatial inequalities alongside socioeconomic disparities provides a more comprehensive understanding of how structural and contextual factors interact to shape the geographic patterns of drug-related deaths. This integrated approach is essential for designing equitable and geographically sensitive public health policies.

 Accordingly, this study aims to analyze spatial inequalities in drug-related mortality across the provinces of Iran in 2020. By identifying areas with high mortality rates and significant disparities, the study will inform the development of targeted prevention and control strategies in order to mitigate the nationwide burden of substance abuse.

## Methods

###  Study design 

 This study employed a cross-sectional ecological design to examine spatial inequalities in drug-related deaths among the provinces of Iran in 2020. The analysis combined epidemiological mortality measures with spatial statistical methods to identify geographic disparities in substance-related deaths. This design is commonly used in spatial epidemiology to evaluate health inequalities at the regional level.^13^

###  Data Sources and Validation

 Mortality data in 2020 were obtained from the Iranian Legal Medicine Organization (LMO), which serves as the national reference for confirming external causes of death and encompasses all forensically confirmed drug-related deaths. The confirmation process at LMO integrates autopsy findings, toxicology reports, and scene investigations, thereby ensuring a high level of diagnostic accuracy. The causes of death were then classified based on the International Classification of Diseases (10^th^ revision; codes T40, T43, F10–F19, X42, X62, and Y12), representing deaths attributed to psychoactive substances and poisoning.^14^ This coding was performed by specialized experts at LMO, adhering to the World Health Organization (WHO) protocols. To ensure data quality, mortality records underwent multi-step validation at the Iranian LMO. Each case was verified through cross-checking autopsy results, toxicology findings, and police or forensic investigation reports. In addition, cases with incomplete, ambiguous, or conflicting information regarding the cause of death were subjected to expert review and recoding according to WHO guidelines. It should be noted that records that remained indeterminate after verification were excluded from the final dataset to prevent potential misclassification bias. Moreover, the LMO applies routine logical consistency checks and periodic data audits in order to maintain the integrity and completeness of its national mortality database. To ensure data reliability, the LMO employs a rigorous quality control process, including logical consistency checks and periodic coding audits. Additionally, provincial population statistics for mortality rate denominators were sourced from the GBD study to ensure consistency and comparability with international epidemiological estimates. All inequality and spatial analyses were performed using age-standardized mortality rates based on the WHO World Standard Population. The human development index (HDI) values for provinces in 2020 were retrieved from the Global Data Lab website (https://globaldatalab.org/shdi/table/shdi/IRN/?levels=1+4&years=2022+2005&interpolation=0&extrapolation=0). Furthermore, geospatial shapefiles for provincial boundaries were acquired from the National Cartographic Center of Iran in order to support spatial analyses. All data were aggregated at the provincial level, and no individual-level identifiers were included from the investigation.

###  Spatial and descriptive data of the study Area

 Iran comprises 31 provinces with diverse geographic, demographic, and socioeconomic characteristics. Its geographical location, bordering Afghanistan and Pakistan (two major drug producers in the world), places the country at high risk for substance use and trafficking.^1^ Variations in development, healthcare access, and urbanization across provinces may contribute to unequal patterns of drug-related mortality. The study area was mapped using ArcGIS 10.3, and spatial disparities were examined by overlaying mortality data on provincial maps to identify potential clusters of high or low mortality.

###  Target and study population

 The target population included all individuals in Iran whose cause of death in 2020 was confirmed to be substance-related, as determined by toxicology and forensic assessments. A census approach was applied, including all eligible cases reported by the LMO without sampling. In addition, mortality was analyzed at the provincial level, with each province serving as the unit of observation for spatial and inequality analyses.^14^

###  Ethical considerations

 Drug abuse-related deaths data were received in an aggregated, anonymized form by the Iranian LMO. Provincial population data in 2020 were retrieved from the GBD database, which is openly accessible from https://vizhub.healthdata.org/gbd-results/. No individual-level identifiers were accessed or analyzed. It is noteworthy that data collection and use followed national legal frameworks governing public health and mortality statistics. Moreover, the study protocol was reviewed and approved by the Ethics Committee of Hamadan University of Medical Sciences (approval No. IR.UMSHA.REC.1404.674). Further, permission to access and analyze the LMO dataset was obtained through formal data-sharing agreements with the organization. All procedures were conducted in accordance with the ethical standards of the Declaration of Helsinki and relevant national guidelines.

###  Statistical analysis

 All analyses were conducted using Stata (version 17) and ArcGIS (version 10.3). Descriptive statistics (means, standard deviations, and frequencies) were calculated for provincial mortality rates. Three indices were applied to evaluate interprovincial inequality in drug-related mortality. The Theil index is a relative inequality measure sensitive to differences across units without natural order ^15^. In addition, the between-group variance (BGV) is an absolute measure of inequality that reflects dispersion from the national average.^15^ Additionally, the relative concentration index (RCI) and concentration curve (CC) are used to examine the distribution of mortality in relation to socioeconomic indicators (e.g., the HDI).^16^ The RCI quantifies the degree of inequality in the distribution of a health outcome (e.g., the mortality rate from drug abuse) based on socioeconomic status. In our study, the HDI was used as a proxy for the socioeconomic status of each province. The RCI is derived from the CC and provides a single number representing the extent of inequality. An RCI value of 0 implies perfect equality. Positive and negative values indicate concentrations of outcomes among wealthier and poorer groups, respectively. The index is calculated as twice the area between the CC and the line of equality, standardized by the total area under the line of equality. Another point about RCI is that its values typically range from -1 to 1. In addition, the CC is a graphical representation showing the cumulative distribution of a health outcome across different socioeconomic groups. The x-axis plots the cumulative percentage of the population ranked by HDI (from lowest to highest), and the y-axis displays the cumulative percentage of mortality from drug abuse. The 45-degree line in the CC demonstrates perfect equality; in this case, every socioeconomic group has an equal share of deaths from drug abuse. The CC depicts how concentrated the outcome is among wealthier or poorer groups. If the curve lies below the line of equality, the outcome is concentrated among wealthier provinces with a higher HDI. Conversely, the outcome is concentrated among poorer provinces with a lower HDI if the curve lies above the line.

 These indices were selected because they capture different but complementary aspects of inequality. The Theil index and BGV are particularly appropriate for measuring disparities across groups that have no natural ordering or hierarchy (e.g., provinces, counties, countries, or demographic subgroups, including gender, age, ethnicity, and race) since they quantify variability in outcomes between such units without assuming any socioeconomic ranking or inherent superiority. In contrast, the RCI is designed for assessing inequality among groups that possess an intrinsic order (e.g., socioeconomic strata, income quintiles, or deprivation levels). The RCI, therefore, reflects the extent to which mortality is disproportionately concentrated among more or less advantaged socioeconomic groups. Overall, these indices provide a comprehensive and multidimensional assessment of both the magnitude and direction of spatial and socioeconomic disparities in drug-related mortality.

 Spatial patterns were assessed using two analysis techniques. Global Moran’s I was utilized to detect overall spatial autocorrelation, and Local Moran’s I (LISA) was used to identify high-high and low-low clusters, as well as spatial outliers. Statistical significance was set at *P* < 0.05. All analyses were weighted by population size to ensure comparability between provinces of different sizes. Furthermore, the spatial weight matrix (W) was constructed using a first-order queen contiguity criterion, where provinces sharing either a common border or vertex were considered neighbors. A binary weighting scheme (1 = neighbor and 0 = non-neighbor) was applied, and the matrix was row-standardized to ensure comparability across provinces with different numbers of neighbors. This matrix was employed for both Global Moran’s I and LISA analyses.

## Results

###  Clinical and perimortem characteristics

**Figure 1:**
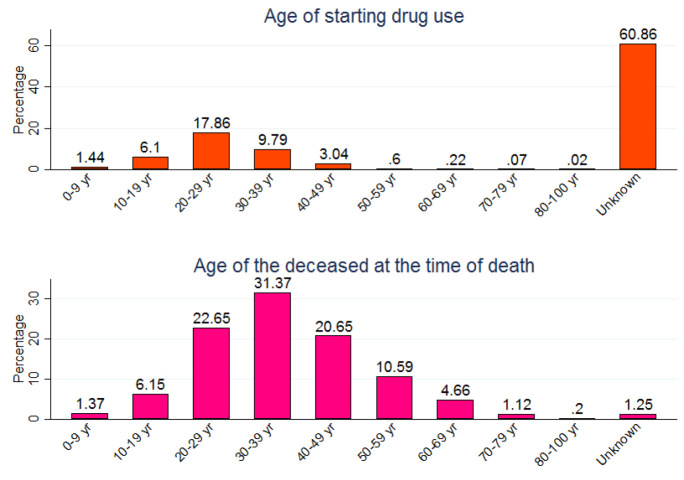
Relative frequency distribution of age: **(A)** age at death and **(B)** age at the onset of drug use among those died from drug abuse in 2020

 A total of 4,637 drug-related deaths were registered in LMO during the study period. Of those, 4,014 cases (86.56%) were included in the present analysis. The majority of the deceased were male (n = 3,446; 85.85%), while females accounted for 568 cases (14.15%). Moreover, 3,792 individuals were identified (94.47%), while 222 cases (5.53%) remained unidentified. Iranian nationals accounted for 94.92% of the deaths, followed by Afghan nationals (2.91%). Other nationalities, including Iraqi and Pakistani, were less than 0.2% each. The 30–39 age group was the most affected (n = 1,259, 31.37%), followed by the 20–29 age group (n = 909, 22.65%) and the 40–49 age group (n = 829, 20.65%). Additionally, individuals younger than 20 years old and those aged 60 years and older represented 302 cases (7.52%) and 240 cases (6.8%), respectively (Figure 1A). Information on the age at which individuals first used drugs was available for 1,572 people (Figure 1B). The mean age of initiation was 25.66 years, with a median of 25 years and a mode of 20 years. In addition, the reported minimum and maximum ages were 1 year and 70 years, respectively. Furthermore, the highest prevalence of initiation was found in the 20–29 age group (17.86%). Regarding marital status, 43.72% of decedents were single, and 37.59% were married. Most of them had less than a high school education, with only 12.6% having completed a university education. Concerning employment, the highest proportion of deaths (38.45%) was observed among self-employed individuals (Table 1).

**Table 1:** Demographic characteristics of drug-related deaths in Iran, 2020

**Variables**	**Number**	**Percent**
Gender		
Male	3,446	85.85
Female	568	14.15
Identity		
Identified	3,792	94.47
Unidentified	222	5.53
Nationality		
Iranian	3,810	94.92
Afghan	117	2.91
Iraqi	3	0.07
Pakistani	1	0.02
Unknown	80	1.99
Other	3	0.07
Living arrangement		
Lived alone	647	16.12
Did not live alone	3,125	77.85
Unknown	242	6.03
Marital status		
Single	1,755	43.72
Married	1,509	37.59
Divorced	504	12.56
Widowed	37	0.92
Unknown	209	5.21
Educational level		
Illiterate	788	19.63
Basic literacy	36	0.90
Primary school	557	13.88
Middle school	1,083	26.98
High school diploma	1,044	26.01
Associate degree	121	3.01
Bachelor’s degree	194	4.83
Master’s degree or higher	47	1.17
Unknown	144	3.59
Occupational status		
Self-employed	1,606	40.01
Student	676	16.84
University student	57	1.42
Unemployed	527	13.13
House wife	335	8.35
Unskilled worker	271	6.75
Skilled worker	28	0.70
Employee	89	2.22
Military personnel	17	0.42
Farmer	31	0.77
Other	250	6.23
Unknown	127	3.16

 Based on the results (Table 2), opium and its derivatives accounted for the largest proportion of deaths (n = 712, 24.89%), followed by methamphetamine (n = 501, 21.23%) and heroin (n = 407, 16.58%). Tramadol (n = 160, 5.92%) and cannabis/multi-drug intoxications (n = 76, 2.73%) contributed to a smaller percentage of cases. Drug abuse was also associated with an increased risk of suicidal ideation, suicide attempts, and completed suicide. The majority of deaths (44.59%) occurred at home. The analysis of living conditions showed that 74.19% of decedents lived in their own homes, while 2.04% were homeless one month before death. At the time of death, 77.85% were not alone, 16.12% lived alone, and 6.03% had unknown living arrangements (Table 2).

**Table 2:** Study of characteristics related to the type of drug, frequency of drug use one month before death, place of death, and place of residence one month before death in drug abuse deceased in 2020

**Variables**	**Number**	**Percent**
Type of drug		
Opium or opium residue	712	24.89
Heroin	407	16.58
Crack	73	2.62
Methamphetamine	501	21.33
Alcohol	375	15.08
Methadone	829	40.80
Tramadol	160	5.92
Other	76	2.73
Number of drugs/alcohols		
0	1,153	28.72
1	2,158	53.76
2	446	11.11
3	158	3.94
4	54	1.35
5	15	0.37
6	14	0.35
≥ 7	16	0.39
Place of death		
Home	1,790	44.59
Prison/detention center	77	1.92
Addiction treatment camp	49	1.22
Harm reduction center	2	0.05
Hospital	1,268	31.59
Unknown	117	2.92
Homeless shelter	18	0.45
Other	245	6.10
Place of residence		
Homeless	82	2.04
Addiction treatment camp	30	0.75
Recovery house	4	0.10
Homeless shelter	7	0.17
Student dormitory	2	0.05
Rental house	401	9.99
Non-student dormitory	2	0.05
Private house	2,978	74.19
Prison	84	2.04
Single-person Residence	123	3.06
Unknown	238	5.93
Hotel	2	0.05
Other	63	1.07
Previous suicide attempts		
Yes	164	4.09
No	3,454	86.05
Unknown	396	9.87
Criminal record		
Yes	42	1.05
No	3,972	98.95

###  Socioeconomic drug abuse mortality rate in Iran (2020)

**Figure 2:**
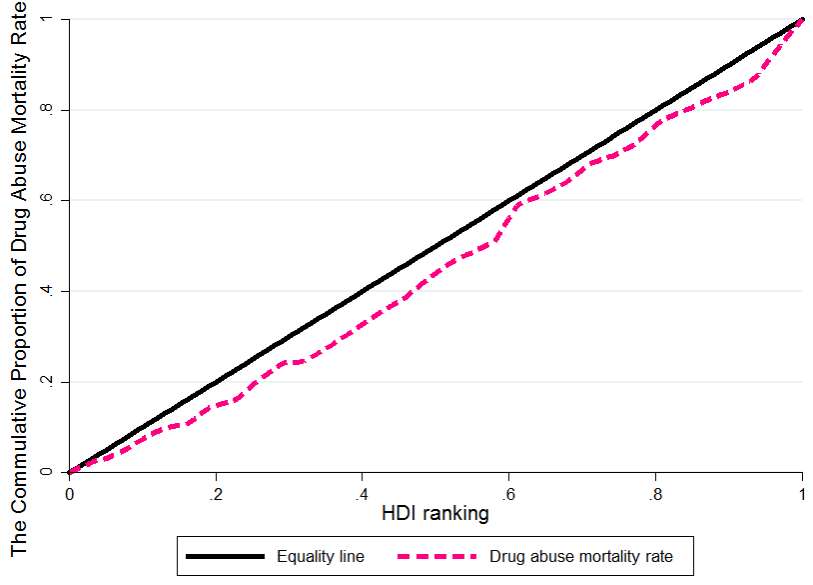
Concentration curve for measuring socioeconomic inequality in the mortality rate of drug abuse in 2020. *Note*. HDI: Human development index

 The RCI was 0.10 for the drug abuse mortality rate (*P* = 0.049). The positive and significant values of the RCI, as well as the placement of the CC below the equality line, demonstrated a higher concentration of drug abuse deaths in provinces with a high HDI (Figure 2).It should be noted that while some low-HDI provinces (e.g., Kermanshah and Lorestan) exhibited particularly high mortality rates, the positive RCI reflected the overall distribution across all provinces weighted by population and HDI. In other words, the cumulative contribution of deaths in higher-HDI provinces was slightly higher at the national level, resulting in a positive RCI, even though specific low-HDI provinces remained hotspots. According to Figure 2, 20% of Iran’s population, with the lowest HDI rank, accounted for approximately 17% of drug abuse deaths. On the other hand, 23% of drug abuse deaths were related to 20% of its population with the highest HDI. Likewise, the poorest and richest 30% of Iran’s population accounted for 23% and 38% of Iran’s drug abuse deaths, respectively.

**Figure 3:**
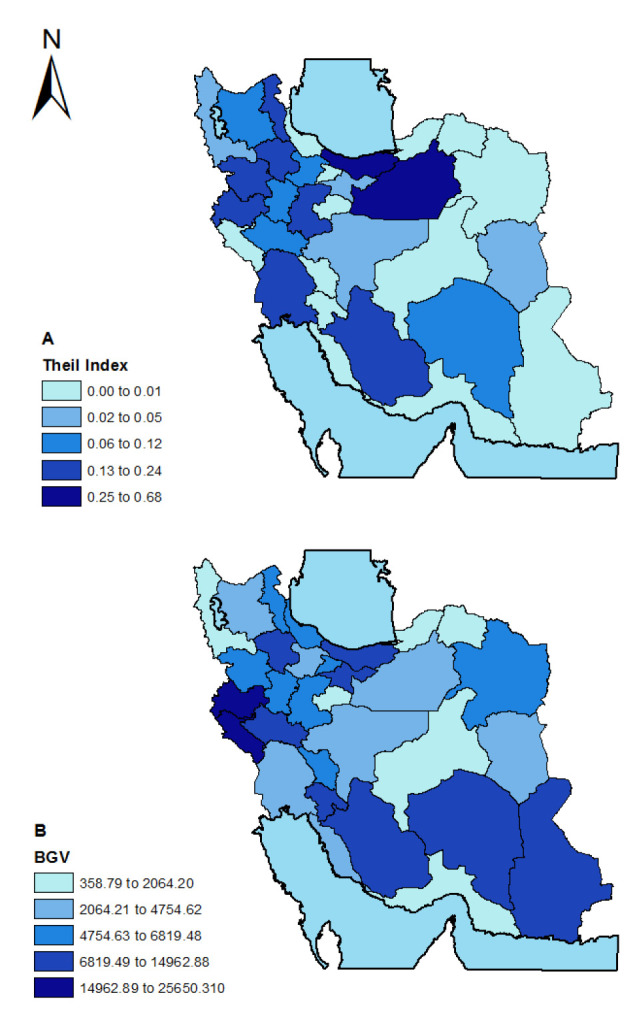
Geographical inequality in mortality rates due to drug abuse in Iran (2020): analysis using **(A)** Theil index and **(B)** BGV. *Note*. BGV: Between-group variance

 The Theil index values calculated for provinces of Iran revealed varying degrees of within-province inequality in drug overdose mortality. Mazandaran (0.673) and Semnan (0.558) had the highest levels of inequality in mortality rate distribution among their counties, indicating a pronounced concentration of drug overdose mortality in specific counties compared to others within these provinces. Conversely, values close to or equal to zero in provinces such as Alborz, Ilam, Bushehr, Qom, Golestan, Gilan, Hormozgan, Sistan and Baluchistan, and Kohgiluye and Boyer-Ahmad suggest a uniform distribution of drug-related deaths across their counties. This pattern implies that all counties within these provinces are affected by the issue of drug overdose mortality at a relatively similar intensity (Figure 3A).

 The BGV index indicated absolute inequality in mortality rates among different provinces within a country. According to this measure, Kermanshah (25,650.31), Ilam (18,351.93), and Kohgiluye and Boyer-Ahmad (14,962.88) had the highest BGV values. These high BGV values signify that the drug overdose mortality rate in these provinces considerably exceeds the national average, identifying them as critical hotspots with exceptionally high mortality rates. In contrast, the provinces of Golestan (358.79), Qom (1,285.56), and Hormozgan (1,024.65) had the lowest BGV values. These provinces have mortality rates much closer to the national mean, indicating a considerably lower intensity of the crisis compared to high-risk provinces (Figure 3B).

###  Drug abuse mortality distribution in Iran (2020)

**Figure 4:**
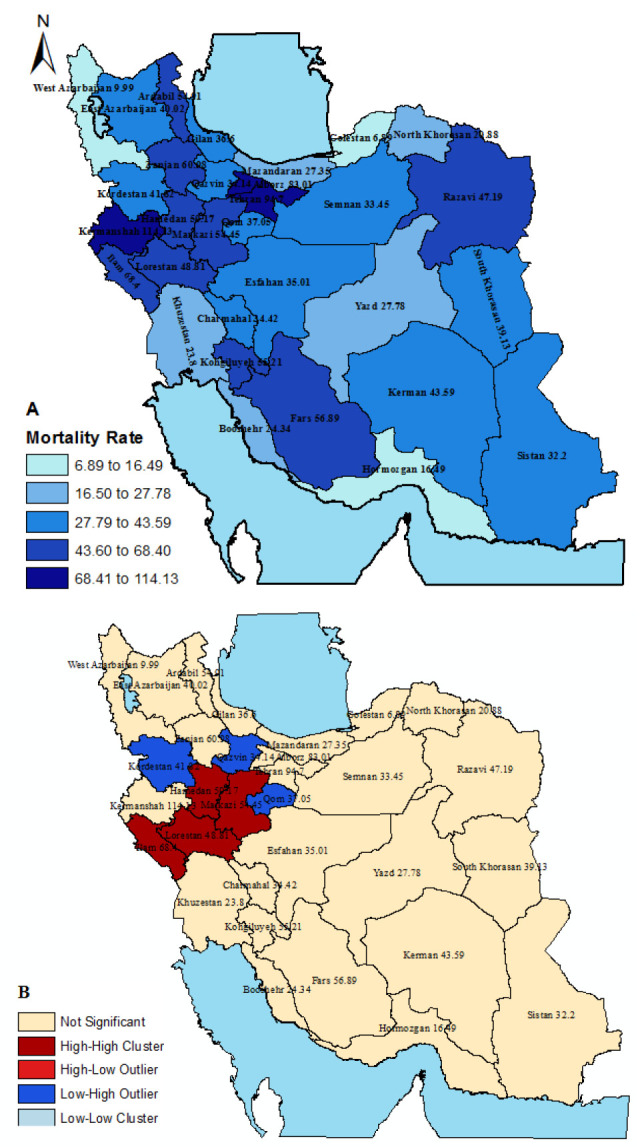
**(A)** Drug abuse mortality rate per 1,000,000 population by provinces in 2020 and **(B)** The clustering map of mortality rates due to drug abuse in the provinces of the country using the Anselin Local Moran I index

 In 2020, the drug abuse mortality rate was 49.91 per million people in Iran. Golestan (6.89 per million people), West Azerbaijan (9.99), Hormozgan (16.49), North Khorasan (20.88), and Khuzestan (23.80) were the provinces with the lowest rates of drug abuse deaths. Conversely, Kermanshah (114.13), Tehran (94.70), Alborz (83.01), Ilam (68.40), and Zanjan (60.98) had the highest rates of drug overdose deaths per million people (Figure 4A).

 Spatial analysis revealed a random overall distribution of drug-related deaths across the provinces of Iran. Global Moran’s I indicated no significant positive spatial autocorrelation (*P* = 0.355), suggesting that provinces with high mortality rates were not clustered geographically. Local Moran’s I (Anselin) analysis identified several significant clusters and outliers. High-high (H-H) clusters were observed in Hamedan, Markazi, Lorestan, and Ilam provinces, indicating high mortality rates surrounded by similarly high-rate provinces. Low-high (L-H) outliers were identified in Kurdistan, Qazvin, and Qom, representing provinces with low mortality located near high-rate provinces. Finally, low-low (L-L) and low-high (L-H) clusters were not observed in mortality rate from drug abuse in 2020 (Figure 4B).

###  Geographical drug abuse mortality rate in Iran (2020)

 Taken together, the Theil and BGV indices confirmed substantial geographic disparities in drug-related mortality across Iran. While inequality within provinces (as captured by the Theil index) varied considerably, the pronounced between-province disparities (reflected in high BGV values) demonstrated that certain western and southwestern provinces are disproportionately burdened by drug overdose deaths, emphasizing the spatially uneven nature of this public health challenge.

## Discussion

 This epidemiological study identified several demographic and socio-economic factors significantly associated with drug-related mortality in Iran. The highest mortality rates were observed among single men aged 30–39 years with low educational attainment and limited income. Additionally, a family history of drug use, previous suicide attempts, and a criminal record were found in varying proportions among the deceased individuals.

 These findings are consistent with those of previous national and regional studies, reporting a disproportionate burden of drug-related mortality among young adult males. For example, a nationwide investigation indicated that 58.5% of illicit drug-related deaths occurred among individuals aged 20–39 years, with 91.4% being male.^17^ The predominance of male mortality in this context may be partly explained by higher mobility, wider social networks, and higher exposure to risky environments among men in Iranian society, whereas lower female mortality rates may reflect stronger familial oversight and cultural restrictions on women’s social interactions.^18^ Nonetheless, the issue of substance use among women should not be underestimated, as it is frequently underreported due to stigma and limited access to treatment facilities. Expanding gender-sensitive rehabilitation services, particularly for women, can improve treatment uptake and provide more reliable epidemiological data on female substance use.^19^

 Dasgupta reported a bimodal age distribution of substance abuse mortality, with the first peak occurring around 40 years of age and the second in the third decade of life.^20^ This pattern closely aligns with the findings of the present study. The predominance of deaths among younger individuals amplifies the years of life lost due to premature mortality and represents a substantial loss of individuals in their productive working years. Similar observations were documented in other epidemiological studies.^21,22^

 In the current analysis, a family history of substance abuse, prior suicide attempts, and a criminal record emerged as significant risk factors. These associations underscore the multifactorial nature of drug-related mortality, in which substance use and adverse social determinants interact to increase vulnerability. In some longitudinal studies, a family history of substance abuse independently increased the risk of SUD in children.^23-26^ The results of the present study also revealed that only 1.05% of the deceased had a criminal history, which is substantially lower than the 39.4% reported by Jalilian et al.^27^ This finding suggests that prior criminal sanctions have not effectively deterred substance use, highlighting the need for revised punitive approaches alongside the implementation of harm reduction strategies. A recent international study reported that lifetime involvement in the criminal justice system was significantly associated with higher odds of SUDs across multiple substances, even after adjusting for sociodemographic and health-related confounders.^28^ A cohort study with a sample size of 122,234 people suggests that young women with a history of prior suicide attempts are at higher risk for subsequent SUD.^29^

 Furthermore, this study identified higher mortality rates among unmarried individuals, which is consistent with the findings of a study performed in Melbourne, Australia, which reported comparable trends.^30^ However, our results contradict the findings of research conducted in Shiraz and Mazandaran provinces in Iran, where lower mortality rates were observed in unmarried individuals. Such discrepancies may be attributable to variations in cultural norms, regional socio-economic conditions, and the strength of social support networks. Nevertheless, these results reinforce the critical role of social support in mitigating the risk of drug-related death, underlining the need for family-based and community-based prevention and intervention programs.^31,32^

 Educational attainment was inversely associated with drug-related mortality, with higher rates observed among individuals with lower levels of education. The increased awareness of psychological, social, economic, familial, and health consequences of substance use may reduce the propensity to engage in drug use. This observation is supported by a cohort study covering 1.6 million subjects conducted in Stockholm, which reported a higher prevalence of substance use among individuals with lower educational levels.^33^ The results of an international study also conform to these results.^34^

 Additionally, most drug-related deaths occurred in homes and other private settings, highlighting the importance of targeted overdose prevention programs for family members, friends, and other close contacts of individuals with SUDs. The effectiveness of interventions that leverage family support in reducing mortality has been documented in previous studies.^30,35,36^

 Employment status emerged as another important factor, with the highest mortality rates found among self-employed individuals, followed by the unemployed and manual laborers. Lack of stable employment and income may increase vulnerability to substance use and related mortality. These findings are in line with those of previous research, documenting similar associations between occupational status and drug-related deaths.^32,37^

 The findings of the Theil index and BGV provided valuable insights into the geographical inequality of drug overdose mortality across the provinces of Iran. The Theil index results indicated significant intra-provincial disparities, with Mazandaran and Semnan provinces exhibiting the highest levels of inequality. This suggests that within these provinces, drug overdose mortality was disproportionately concentrated in a few counties, highlighting the stark regional variation in how different areas are affected by this crisis. In contrast, Alborz, Ilam, and Hormozgan provinces showed near-zero values on the Theil index, reflecting a more even distribution of mortality rates across counties. This implies that the drug overdose issue, while still a concern, is more evenly spread out within these provinces, potentially pointing to a broader, less localized impact. On the other hand, the BGV index revealed inter-provincial inequalities, showing that certain provinces, including Kermanshah, Ilam, and Kohgiluye and Boyer-Ahmad, had significantly higher drug overdose mortality rates than the national average. The elevated BGV values indicate that these provinces are facing an exceptionally severe drug crisis, with mortality rates far above the national mean, indicating a need for targeted intervention and policy response. Meanwhile, Golestan, Qom, and Hormozgan provinces, with much lower BGV values, are experiencing a relatively moderate crisis. These findings highlight the importance of tailoring public health responses to address the specific needs of each province, as the intensity and distribution of the drug overdose issue vary widely across the country. Moreover, although Global Moran’s I indicated no significant spatial autocorrelation at the national level, LISA identified significant clusters in specific provinces. This contrast confirms that while drug-related deaths were spatially scattered across the country overall, certain provinces exhibited localized high-mortality clusters due to contextual and regional factors. Additionally, these observed provincial and intra-provincial inequalities have important implications for resource allocation and public health planning. Provinces with higher Theil and BGV values, demonstrating concentrated high-mortality areas, may require targeted interventions, increased funding for harm reduction programs, and enhanced healthcare services to effectively reduce drug-related deaths. In contrast, provinces with lower inequality indices might benefit more from broad-based preventive strategies and education campaigns. These findings underscore the need for province-specific policy actions and targeted interventions. Critical hotspot provinces, such as Kermanshah, Ilam, and Kohgiluye and Boyer-Ahmad, could benefit from enhanced harm reduction programs, expanded access to treatment facilities, focused community education campaigns, and strategic allocation of healthcare resources. Such targeted measures may help reduce drug-related mortality more efficiently and maximize the impact of public health interventions in areas with the highest burden.

 Finally, this study underscores the need for targeted interventions to reduce drug-related mortality in Iran, particularly among young, unmarried men with low education and income levels. Implementing harm reduction programs, enhancing public education on the risks of substance use, and providing support to families affected by SUDs are essential steps in mitigating the impact of drug-related deaths. Likewise, evidence-based interventions, such as naloxone distribution and methadone maintenance therapy, which have demonstrated effectiveness in numerous studies, should be prioritized.

 As with any research, this study has some limitations. The completeness and reliability of mortality records cannot be fully guaranteed, and information provided by the relatives of the deceased may have been inaccurate or incomplete. Accordingly, addressing these limitations in future research through improved surveillance systems and standardized data collection methods will enhance the accuracy of findings and support more effective policy-making.

**Highlights**

- The mortality rate due to drug abuse in Iran was 49.91 per million people in 2020.
- Spatial clustering patterns highlight high-risk areas in western Iran.
- The positive concentration index indicated a higher concentration of drug-related deaths in provinces with higher HDI.
- The Theil and between-group variance (BGV) indices confirmed the existence of geographical inequality both within and between provinces.

## Conclusion

 Our findings revealed that drug-related deaths in Iran predominantly affect males, with the highest mortality rates observed in the 30–39 age group. It was found that victims often have lower educational attainment and are primarily self-employed. Geographically, provinces with lower human development indices, such as Kermanshah, Hamedan, and Lorestan, demonstrated the highest mortality rates. Thus, these regions require targeted prevention and treatment interventions that consider their unique socioeconomic contexts. Significant geographical disparities in drug-related mortality were observed across Iranian provinces, particularly in Kermanshah, Hamedan, Lorestan, and Fars. These findings highlight the need for region-specific strategies and targeted allocation of resources to enhance the effectiveness of prevention and treatment programs. The most common substances involved in these fatalities are opium and its derivatives, methamphetamine, and heroin. Furthermore, the association between substance use and suicidal behaviors calls for integrated mental health and addiction treatment services. To reduce the public health burden of drug abuse, policymakers should focus on strengthening educational, prevention, and treatment programs for high-risk populations and regions with high mortality rates.

## Acknowledgments

 The authors would like to acknowledge Hamadan University of Medical Sciences for supporting this research.

## Competing Interests

 The authors declare no conflict of interests.

## Ethical Approval

 This study was conducted at Hamadan University of Medical Sciences, Iran. The research protocol was reviewed and approved by the Ethics Committee of Hamadan University of Medical Sciences (Ethical No. IR.UMSHA.REC.1404.674).

## Funding

 This study was financially supported by Hamadan University of Medical Sciences, Iran.
